# Thoracolumbar Scoliosis Due to Cryptococcal Osteomyelitis: A Case Report and Review of the Literature

**DOI:** 10.1097/MD.0000000000002613

**Published:** 2016-02-08

**Authors:** Zheng Li, Jinqian Liang, Jianxiong Shen, Guixing Qiu, Xisheng Weng

**Affiliations:** From the Department of Orthopaedic Surgery, Peking Union Medical College Hospital, Chinese Academy of Medical Sciences and Peking Union Medical College, Beijing, China.

## Abstract

Cryptococcus neoformans causes opportunistic infections in immunocompromised patients, with vertebral osteomyelitis being a very rare involvement.

This study is to present a case of thoracolumbar scoliosis occurring in the setting of cryptococcal osteomyelitis.

Pharmacological intervention with anticryptococcal medicine and medical management of immune hemolytic anemia were administered. After initial acute stabilization, she underwent spinal debridement and fusion on October 29, 2008. She eventually recovered fully from this episode with no subsequent mechanical instability or neurological deficits on subsequent clinic follow-ups.

To the best of our knowledge, there have been no reports describing the onset of spinal cryptococcal osteomyelitis along with immune hemolytic anemia. We suggest a comprehensive algorithm for the diagnosis of vertebral cryptococcal osteomyelitis.

## INTRODUCTION

Cryptococcus was first found in the environment and then in human as cryptococcal osteomyelitis of tibia.^1,2^ Cryptococcosis is classically an opportunistic invasive fungal infection caused by Cryptococcus neoformans predominantly in immunocompromised hosts.^1–3^ It is associated with sarcoidosis, AIDS, steroid therapy, lymphoma, organ transplantation, tuberculosis, and other immune-compromised patient.^1,4,5^ The primary sites are the central nervous system and lungs, eyes, urinary tract, joints, and skin.^6,7^ There are limited reports regarding the diagnosis and management of cryptococcal osteomyelitis with its possible resultant scoliosis. Here, we report a case of cryptococcal osteomyelitis in the thoracolumbar scoliosis of a 17-year-old woman.

### Consent

Written informed consent was obtained from the patient's parents on behalf of the child for publication of this Case Report and any accompanying images. A copy of the written consent is available for review by the Editor of this journal.

### Case Report

**FIGURE 1:**
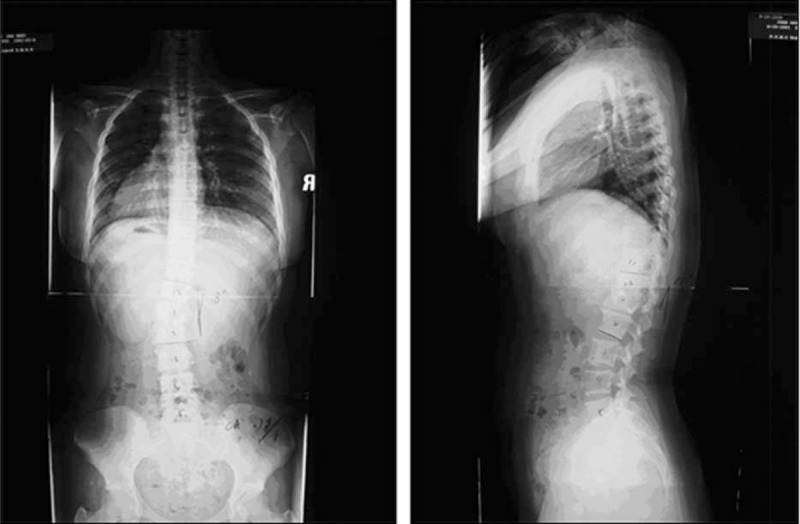
Standing anteroposterior and lateral radiographs of the preoperation.

A 17-year-old patient was initially diagnosed with L1 vertebra tuberculosis and left-sided thoracolumbar scoliosis in August 1, 2007 (Figure 1), and treated with anti-TB medication. Despite a month of anti-TB treatment, the patient continued to be symptomatic with intermittent low-grade fever with maximum temperature of 37.5°C as well as worsening back pain. The fever, which occurred more frequently at night, was accompanied by night sweats and would abate spontaneously. On September 20, the patient developed neutropenia, and a bone marrow biopsy was performed. The result was diagnostic of granulopenia, and Granulocyte-colony stimulating factor (G-CSF) was administered on September 21. The patient's fever worsened with a maximum temperature of 38.5°C, headache, and nausea on November 20. Her clinical picture was very suspicious of CNS tuberculous infection, and lumbar punctures were carried out twice. Cryptococcus Neoformans was isolated in both CSF cultures. Serial titer for cryptococcus antigen was 1:512. 5-FC and fluconazole were administered orally from December 12 and anti-TB medication was withdrawn. CRP returned normal on December 22, and thoracolumbar computed tomography indicated that the surrounding soft tissue swelling had resolved, despite the vertebral destruction remaining status quo. Despite the radiological evidence of resolution of the infection, the patient's fever (37.7°C), headache, dark colored urine, nausea, and intermittent nonprojectile vomiting resurfaced on the February 25 the following year without any attributable causes. Tests for autoantibodies were all negative, while the Coombs’ Test returned positive and a bone marrow biopsy was consistent with immune hemolytic anemia. Hydrocortisone sodium succinate was prescribed, augmented by 400 mg of fluconazole a day and urine alkalization.

**FIGURE 2:**
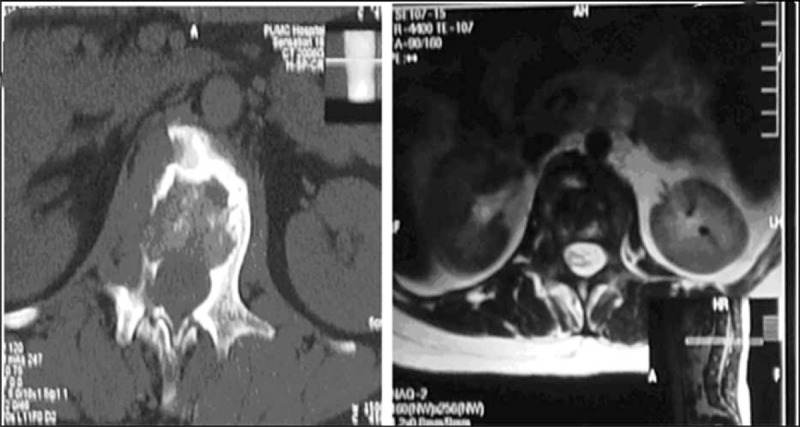
Computed tomography (CT) scan and magnetic resonance imaging (MRI) done in May 2007 collectively pointed toward a left-sided scoliosis with L1 vertebral body destruction resembling tuberculous osteomyelitis.

However, the patient's clinical picture deteriorated acutely 3 days later and she was transferred to the intensive care unit with tachycardia at 120 to 130 bpm, hypotensive with a blood pressure of 90–100/50–70 mm Hg and core body temperature of 39.8°C. Fluid and plasma resuscitative substitution were carried out with 3000 mL infusion of fresh frozen plasma and 1500 mL of physiological saline infusion. Three hours later, the patient exhibited a diminished response to stimulus with a GCS of E2V1M6 (=9). Her hemoglobin level plunged to 12 g/L, while blood typing and cross-matching were repeatedly incompatible. After further consultation with a hematologist, 6 units of abstergent O-type, Rh (+) RBC plus 10 g of IVIG were infused, after which the patient's GCS improved to E4V5M6 (=15). No transfusion reaction was noticed. Three hundred milligram of hydrocortisone plus 10 g of IVIG was administered on February 29, with no further signs of active hemolysis detected. A second plasma substitution, with 2000 mL infusion of fresh frozen plasma and 3050 mL of plasma saline, was performed on March 3 in an attempt to dilute the autoantibodies. The patient was discharged from the ICU on March 3 with an Hb of 75 g/L and total bilirubin of 38.9 μmol/L. A peripheral blood film showed that RBCs were dispersed in size with 21% of spherocytes. Both erythrocyte fragility test and Rous test were positive, confirming the diagnosis of IHA. CsA plus IVIG was administered. Lumbar puncture, ANA, anti-ENA, ESR, CRP, and bone marrow biopsy were repeated with no further significant findings. Roenterography, computed tomography scan, and magnetic resonance imaging had done collectively pointed toward a left-sided scoliosis with L1 vertebral body destruction resembling tuberculous osteomyelitis (Figure 2).

**FIGURE 3:**
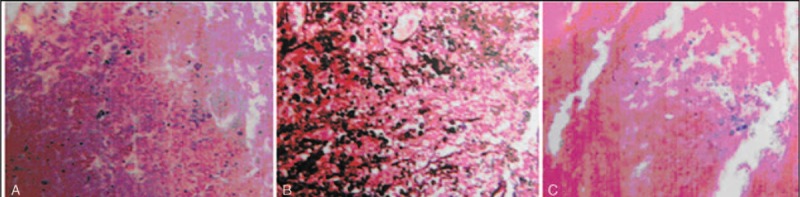
Histo-pathological slides: L1 vertebral body demonstrating necrotic material and granulation tissue with large amounts of cryptococcal organisms on a background of chronic inflammation involving bony and fibrous connective tissue. Special staining: A, Mucin carmine (MC) (+); B, Gomori methenamine silver (GMS) (+); C, Hematoxyline and Eosin (H&E) (+).

The patient underwent thoracolumbar debridement and fusion through posterior approach on October 29, 2008, with intraoperative intravenous titration of dexamethasone sodium phosphate and haemocoagulase under ceftazidime cover. Samples from L1 vertebra and the para-vertebral abscess were sent to the laboratory, and specific stains for Cryptococcus Neoformans (Mucicarmine stain, Gomori's methenamine silver stain, and Hematoxyline and Eosin stain) all returned positive (Figure 3).

**FIGURE 4:**
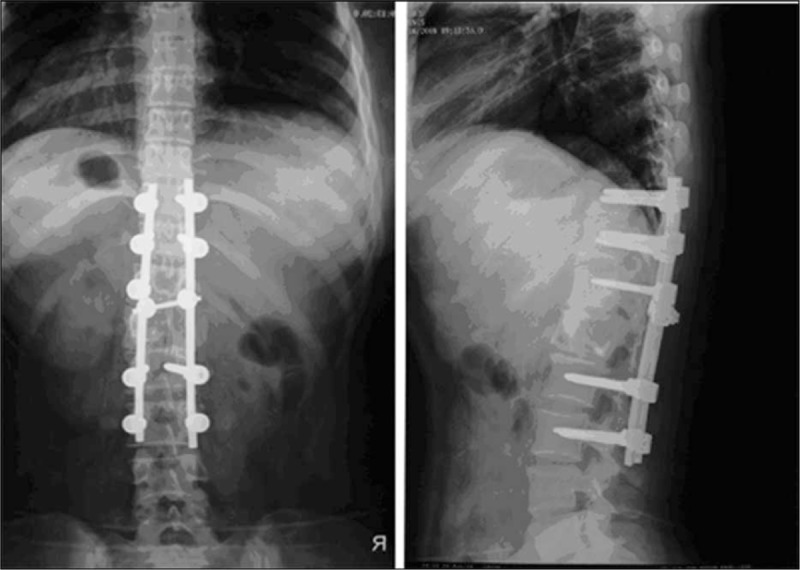
Standing anteroposterior and lateral radiographs of 4 days after operation.

With the definitive intraoperative histo-pathological evidence of Cryptococcus Neoformans infection, postoperative treatment of fluconazole, leucogen tablets, and CsA were administered for prophylactic measures for 3 months. Full recovery in terms of mechanical stability with neurological integrity was achieved in subsequent clinic follow-ups (Figure 4).

## DISCUSSION

Cryptococcus neoformans causes opportunistic infections in immunocompromised patients, typically among patients who are debilitated by cancer or diabetes, subjects undergoing steroid treatment or chemotherapy, and in particular, patients with AIDS.^8,9^ Its occurrence in healthy population is extremely rare, as is vertebral involvement in cryptococcal infection.^10,11^ Cyptococcal infection can spread after initial inhalation into the lung, disseminating to CNS, skin, bone, kidney, liver, spleen, and lymph nodes.^12,13^ Since the patient had recovered from both skin and gum infections after anticryptococcal treatments, the cryptococcal osteomyelitis may be inferred as a result of dissemination from previous infections.^14^ In the present study, we reported a case of cryptococcal osteomyelitis in the thoracolumbar scoliosis of a 17-year-old woman. The patient underwent thoracolumbar debridement and fusion through posterior approach and full recovery in terms of mechanical stability with neurological integrity was achieved in subsequent clinic follow-ups.

The patient had an episode of IHA with findings consistent with Coombs’ Test and bone marrow biopsy, which may be drug-induced hemolysis. Rifampin, for example, causes hemolysis by the formation of an antibody–drug complex and complement activation.^15^ Therefore, drug-induced IHA is a possible postulate.^16^ However, IHA may also be a result of infection. The patient went through a chronic phase of disease since August 2007, and her WBC count was diminished to 0.82 × 109/L on 1 occasion in the interim, both of which heightened her susceptibility to infection. The IHA developed 2 months after withdrawal of anti-TB treatment, all of which point to the lower likelihood of drug-induced IHA as compared with the higher likelihood of postinfection IHA.

Moreover, if we assume that IHA were to precede the infection, we can hypothesize that IHA may be a predisposing factor toward cryptococcal osteomyelitis susceptibility. Proteus mirabilis, Klebsiella pneumoniae, and Salmonella osteomyelitis have been reported in sickle cell anemia and evidence for HLA class II association with osteomyelitis as a complication of sickle cell anemia has been well established. With this reference, we extra-polate that cryptococcal osteomyelitis may also be a complication associated with IHA. However, no report has been published so far suggesting this association and relationship.

## CONCLUSION

To the best of our knowledge, there have been no reports describing the onset of spinal cryptococcal osteomyelitis along with IHA. We recommend that microscopic examination and microbial cultures can be a good option employed to definitively identify the pathogen as well as its drug sensitivity. We also suggest the above algorithm using magnetic resonance imaging as well as microbiological analysis as a comprehensive method to accurately diagnose vertebral cryptococcal osteomyelitis.
